# Direct oral anticoagulants for treatment of deep vein thrombosis: overview of systematic reviews

**DOI:** 10.1590/1677-5449.005518

**Published:** 2018

**Authors:** Gustavo Muçouçah Sampaio Brandão, Raissa Carolina Fonseca Cândido, Hamilton de Almeida Rollo, Marcone Lima Sobreira, Daniela R. Junqueira

**Affiliations:** 1 Universidade Federal de São Carlos – UFSCar, Departamento de Medicina, Saúde do Adulto e Idoso - Cirurgia, São Carlos, SP, Brasil.; 2 Universidade Federal de Minas Gerais – UFMG, Centro de Estudos do Medicamento, Departamento de Farmácia Social, Faculdade de Farmácia, Belo Horizonte, MG, Brasil.; 3 Universidade Estadual Paulista “Júlio de Mesquita Filho” – UNESP, Faculdade de Medicina de Botucatu, Departamento de Cirurgia e Ortopedia, Botucatu, SP, Brasil.; 4 University of Alberta, Edmonton, Canada.

**Keywords:** direct oral anticoagulants, deep venous thrombosis, systematic review

## Abstract

A number of limitations of standard therapy with warfarin for deep vein thrombosis (DVT) have been established. This overview of systematic reviews presents the baseline results for efficacy and safety of the new direct oral anticoagulants (DOACs) thrombin inhibitors, and activated factor X (Xa) inhibitors in patients with DVT. Searches were run on PubMed and the Cochrane Database of Systematic Reviews. Twenty-three studies were retrieved, and one systematic review was judged eligible. This review scored maximum according to AMSTAR criteria and included 7,596 patients for analysis of thrombin inhibitors and 16,356 patients for analysis of factor Xa inhibitors. The results of the meta-analysis indicate that DOACs are similar for DVT treatment when compared to standard treatment with warfarin. The incidence of major bleeding is somewhat lower in patients treated with factor Xa inhibitors and similar to standard therapy when treated with direct thrombin inhibitors.

## INTRODUCTION

 Deep venous thrombosis (DVT) in the lower limbs is a serious and potentially fatal disease. Incidence in the general population is five cases per 10,000 inhabitants annually. 1 Approximately 46% of proximal DVT cases (i.e. DVT involving the illeo-femoral, femoral, and popliteal regions) can progress to pulmonary embolism (PE), an event which, if not treated, is fatal in 4% of cases. 2 Furthermore, important complications, such as postthrombotic syndrome, can occur in up to 50% of patients who suffer DVT. 3 Thus, after confirmation of a DVT diagnosis, it is imperative to start anticoagulant treatment. The objective of treatment is to relieve symptoms, reduce the extent of the thrombus and the likelihood of a PE, prevent recurrence, and attenuate postthrombotic syndrome. 

 Standard treatment is initially based on parenteral administration of unfractionated heparin or low molecular weight heparin for 5 to 7 days, followed by long-term treatment with oral vitamin K antagonists (VKAs). 4 ^,^ 5 Like warfarin, VKAs have traditionally been used as oral anticoagulants for treatment and prophylaxis of venous thromboembolism (VTE) since the 1950s. 6 ^,^ 7 Despite its efficacy, use of warfarin is limited by factors such as drug interactions, food interactions, slow onset of action, risk of hemorrhage, alopecia, skin necrosis, and a need for rigorous monitoring to maintain the international normalized ratio (RNI) within the therapeutic range. These limitations have driven research into new anticoagulants which, ideally, should offer a reduced risk of bleeding and reduced rate of side effects, be free from interactions with other medications and foods and easy to administer, enable home treatment, not require laboratory tests for control, be inexpensive, and have an antidote to reverse anticoagulation in case of extensive and clinically relevant bleeding. 8

### Direct oral anticoagulants

 According to the American College of Chest Physicians (ACCP) guidelines, 9 two forms of oral anticoagulants are indicated for treatment of DVT: direct thrombin inhibitors and factor Xa inhibitors. 

 Direct thrombin inhibitors, such as dabigatran, bond directly to thrombin with no need for a cofactor, such as antithrombin. Unlike VKAs and heparins, direct thrombin inhibitors can inhibit both soluble thrombin and thrombin bound to fibrin. 10 Since they do not bind to other proteins, direct thrombin inhibitors have few pharmacokinetic and pharmacodynamic restrictions, which makes the anticoagulant response more predictable. Additionally, direct thrombin inhibitors do not have an antiplatelet effect and do not induce the thrombocytopenia induced by heparin. 11

 Factor Xa inhibitors bond directly to the active site of factor Xa, blocking this coagulation factor’s activity. In contrast to the pentasaccharides (indirect factor Xa inhibitors), these medications inactivate free factor Xa and factor Xa that is has been incorporated into complex prothrombinase, and do not interact with antithrombin inhibitor. 12 The factor Xa inhibitors that are indicated for treatment of DVT are: rivaroxaban, apixaban, and edoxaban. 

 Safe and effective clinical use of direct oral anticoagulants (DOACs) demands monitoring of evidence of their clinical efficacy. Studies of their adverse effects, primarily bleeding, are also an essential part of understanding the balance between benefits and harmful effects in comparison with the anticoagulants currently used in clinical practice. Our objective was therefore to review the scientific evidence on the benefits and adverse effects of new anticoagulants for treatment of DVT compared with standard treatment (low molecular weight heparin or unfractionated heparin followed by VKAs). 

## METHODS

 This is an overview of systematic reviews of the efficacy and adverse effects of treating patients with DVT using DOACs, selecting studies that systematically review controlled and randomized clinical trials comparing the standard treatment with DOACs in patients diagnosed with DVT. No publication date or language restrictions were imposed. Narrative reviews, guidelines, and specialist opinions were not included. 

 Searches were run on PubMed and the Cochrane Database of Systematic Reviews. The search run on the PubMed database employed a high-sensitivity search filter to return systematic reviews. 13 The search strategy used for both databases employed a combination of the terms with their respective truncation symbols: novel oral anticoagulant* *and deep vein thrombosis* . 

 The studies retrieved were selected independently by two authors. Initial screening consisted of reading all titles and abstracts and then the full texts of articles considered potentially eligible in the first stage were analyzed. Disagreements during the selection process were resolved by participation of a third author. 

 Two outcomes were considered essential for assessment of efficacy and adverse effects of DOACs in relation to VKAs: (i) recurrence of DVT or VTE; and (ii) bleeding. Data on these outcomes and a general description of studies were extracted by one author and reviewed by a second author. Data were extracted using a data collection form, describing studies according to the update date of the systematic search, the patient population studied, the type of oral anticoagulant studied, and the number of clinical trials included. 

 The internal validity (methodological quality) of systematic reviews was assessed using the AMSTAR tool. 14 ^,^ 15 AMSTAR is a validated tool comprising 11 items with direct responses (Yes, No, Can’t answer, and Not applicable) which evaluates, among other elements, existence of a priori planning of the systematic review, whether selection and extraction of data was conducted in duplicate, and whether status of publication was used as an inclusion criterion. The quality of a review can be analyzed on the basis of the final score (a maximum of 11 points). 

 The systematic reviews included were described in terms of their general characteristics (for example, patient populations, medications studied), results of outcomes of interest, and methodological quality. Statistical analysis (meta-analysis) was not possible because of the limited number of studies included. 

## RESULTS

**Figure 1:**
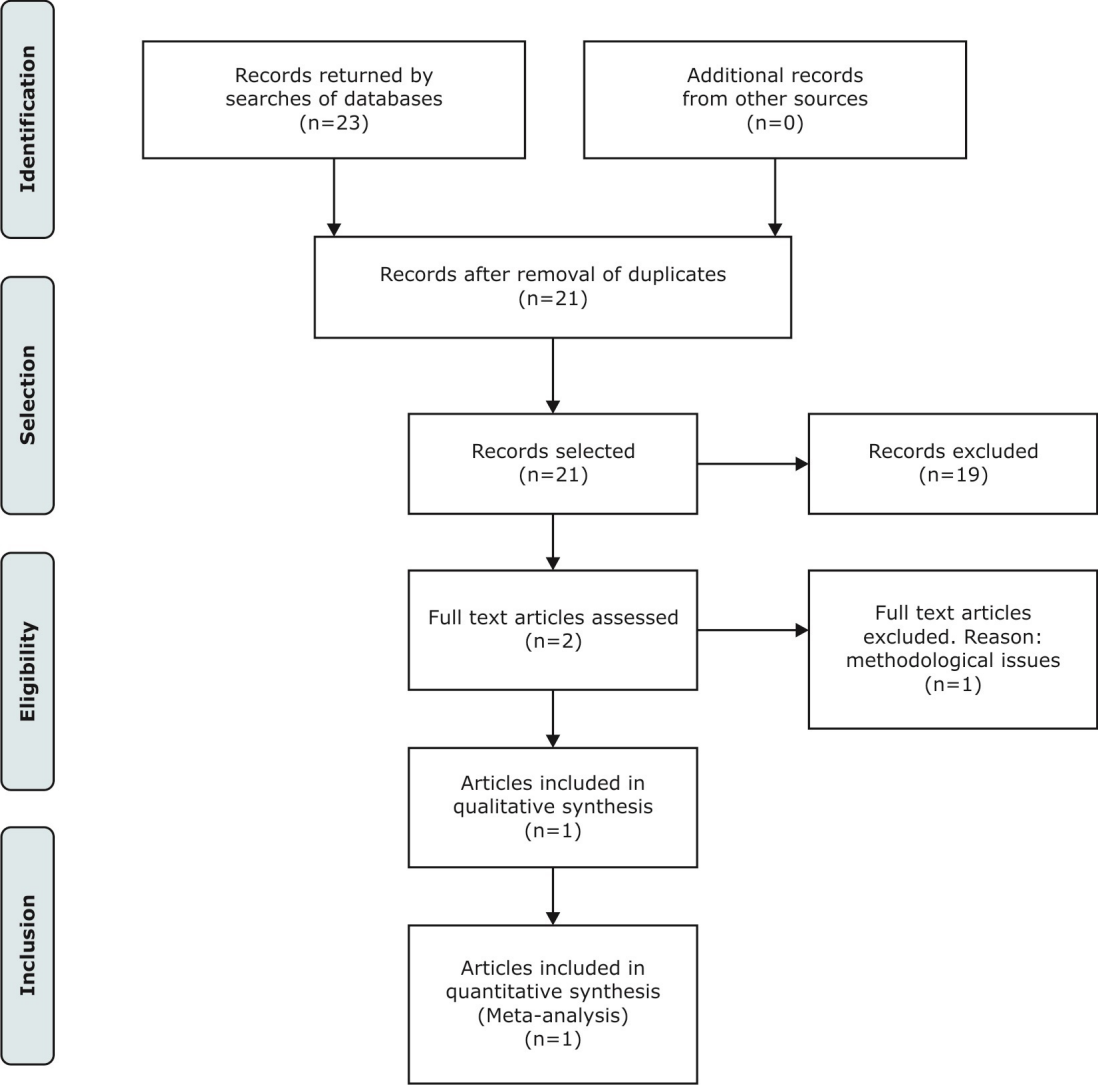
Diagram illustrating identification and selection of articles for systematic review comparing standard treatment of deep venous thrombosis with new oral anticoagulants.

 A total of 23 articles were retrieved by the searches. After exclusion of duplicates, 21 articles were assessed against the inclusion criteria. Of these, 19 articles were excluded after reading titles and abstracts, and the full texts of two articles that were considered potentially eligible were analyzed. At the end of the selection process, one systematic review was considered eligible and included in our review ( Figure 1 ). 

 A systematic review conducted by Robertson et al. 16 compared treatment with direct inhibitors of thrombin and factor Xa with standard treatment. All thrombin inhibitors and all factor Xa inhibitors were compared as a single group, and individual comparisons for each medication in the two classes were not conducted. The comparison of thrombin inhibitors included one study using the medication ximelagatran, which was withdrawn from the market in 2006 because of reports of severe liver damage with continuous use (more than 11 days). 17

 This review scored maximum points on AMSTAR ( Table 1 ) and covered 7,596 patients for analysis of direct thrombin inhibitors and 16,356 patients for analysis of direct factor Xa inhibitors ( Table 2 ). The results of a meta-analysis conducted in this systematic review indicated that efficacy for prevention of venous thromboembolism and incidence of major bleeding (severe hemorrhage meeting the International Society of Thrombosis and Hemostasis [ISTH] definition 18 ) was similar for standard treatment and oral anticoagulants in the direct thrombin inhibitor class. Factor Xa inhibitor DOACs also exhibited similar efficacy to standard treatment, while the incidence of major bleeding was a little lower for patients given factor Xa inhibitor DOACs. 

**Table 1:** Description of the systematic review comparing standard treatment for deep venous thrombosis with new oral anticoagulants.

**Author, Year**	**Search date**	**Population**	**Oral anticoagulants**	**Clinical trials included (n)**	**AMSTAR score**
Robertson, 2015	January 2017	Patients with diagnosis of deep venous thrombosis confirmed by standard imaging technique (venography, plethysmographic impedance, distal compression ultrasonography, proximal compression ultrasonography)	Direct thrombin inhibitors and direct factor Xa inhibitors	3 clinical trials comparing direct thrombin inhibitors with standard treatment; 8 clinical trials comparing direct factor Xa inhibitors with standard treatment	11/11

**Table 2:** Recurrence of venous thromboembolism and incidence of major bleeding in patients with deep venous thrombosis treated with new oral anticoagulants and comparison with standard treatment.

**Comparison**	**Patients**	**Recurrence of deep venous thrombosis or venous embolism (OR, 95%CI)**	**Major bleeding**	**Quality of studies contributing to meta-analysis**
Direct thrombin inhibitors (ximelagatran, dabigatran)	7,596 patients, mean age (min 54.7, max. 57.1)	≤ 3 months: OR 1.09 (95%CI 0.62-1.91);	≤ 3 months: (OR 0.54; 95%CI 0.28-1.03);	Authors’ judgment: “*We deemed all included studies to be of high methodological quality and generally low risk of bias*.”
		> 3 months: OR 1.09 (95%CI 0.76-1.58);	> 3 months: (OR 0.76; 95%CI 0.49-1.18);	
		6 months: 1.09 (95%CI 0.80-1.49)	6 months: OR 0.68 (95%CI 0.47-0.98)	
Direct factor Xa inhibitors (apixaban, rivaroxaban, edoxaban)	16,356 patients mean age (min. 53.1, max. 60)	≤ 3 months: OR 0.69 (95%CI 0.48-0.99);	≤ 3 months: OR 0.83 (95%CI 0.47-1.45);	Authors’ judgment: “*We deemed all included studies to be of high methodological quality and generally low risk of bias*.”
		> 3 months: OR 0.97 (95%CI 0.78-1.22);	> 3 months: OR 0.50 (95%CI 0.36-0.71);	
		6 months: 0.89 (95%CI 0.73-1.07)	6 months: OR 0.57 (95%CI 0.43-0.76)	

OR: odds ratio; CI: confidence interval.

## DISCUSSION

 The accumulated evidence indicates that DOACs, both direct thrombin inhibitors and direct factor Xa inhibitors, offer a balance between efficacy and adverse effects similar to standard treatment. This means that these medications do not offer a different balance between risk and benefit than the combination of heparins and VKAs for treatment of DVT. 

 The evidence found is limited to a single systematic review. The systematic review available for this evaluation was considered of high methodological quality. However, the likelihood of its results being modified by future research depends on the methodological quality of the clinical trials included and analyzed by the systematic review. Unfortunately, the systematic review in question did not conduct an assessment of evidence quality using the Grading of Recommendations, Assessment, Development, and Evaluations (GRADE) methodology, 19 ^-^ 21 which would have made it possible to infer with greater confidence the degree of quality of this evidence and the clinical recommendations in force. The number of patients included in the quantitative analysis of the outcomes recurrence of DVT or PE and major bleeding was considerable. However, the quality of the randomized clinical trials included in the systematic review appears to suffer from risk of bias, primarily in relation to generation of the sequence of random allocation to treatment. Thus, while the conclusions appear consistent, additional evidence would increase the reliability of the data for making definitive clinical decisions. 

 One limitation of these results is the fact that analysis of efficacy and adverse effects was only conducted taking medications in each of the two DOAC categories, direct thrombin inhibitors and direct factor Xa inhibitors, together as a group. While it is probable that individual medications that share the same mechanism of action will exhibit similar effects, it is useful to confirm the clinical effects of each medication empirically. 

 The DOACs offer simpler management of anticoagulant treatment; they are administered in fixed doses with no need for adjustment by body weight. These medications have rapid onset of action, do not need to be controlled using laboratory test results, and have a short half-life (facilitating patient management when there is a need to suspend medications to conduct a diagnostic or surgical procedure). The drug-drug, drug-alcohol, and drug-food interactions described to date are few and permit use in ambulatory level treatment. 

 One practical problem with use of DOACs, however, is related to these medications’ different treatments, doses and, primarily, posologies. 

 This is because, while some are used in monotherapy (for example, rivaroxaban and apixaban), others (for example, dabigatran and edoxaban) are used as an adjuvant therapy with low molecular weight heparin. Additionally, even medications used as monotherapy have different doses and posologies, which may vary during the initial phase and maintenance phase of anticoagulation (for example, rivaroxaban). The standard treatment, which is established clinical practice for more than 50 years, offers a therapeutic regimen that is more effective, safe, and compatible with care at the ambulatory level, despite the need for dosage adjustments. Another relevant practical problem is access to these new medications, especially in countries with low levels of socioeconomic development, since they are expensive. 

 One determinant factor for routine clinical use of DOACs is availability of a reversal agent for the entire class of medications. The anticoagulant effect of VKAs can be reversed by administration of vitamin K, fresh frozen plasma, or prothrombin complex. 22 Idarucizumab, a reversal agent specifically for dabigatran, has already been approved for clinical use in the United States. It is a fragment of monoclonal antibody which has a greater affinity for dabigatran than thrombin and therefore reinstates the coagulatory effect. 23 Andexanet alfa, a recombinant factor Xa molecule, which can bind both to direct factor Xa inhibitors (such as rivaroxaban, apixaban, and edoxaban) and to factor Xa inhibitors that require antithrombin activity (such as low molecular weight heparin and fondaparinux), was also approved recently. 24 ^,^ 25 In Brazil, only idarucizumab has already been authorized for use and sale. 26

 Below, we present a summarized review of the status of available phase III studies of the different DOACs. 

### Dabigatran

 Dabigatran etexilate (Pradaxa®) is a pro-drug that is rapidly metabolized by the liver, transforming it into an active compound that binds competitively and reversibly to the thrombin site of activity, blocking its procoagulatory activity. Dabigatran is absorbed by the gastrointestinal tract, has a half-life of 12 to 17 hours, and renal and fecal excretion. The RE-COVER study compared treatment with warfarin to dabigatran, after initial treatment with a parenteral anticoagulant, in 2,539 patients diagnosed with acute VTE over a 6-month period. 27 The results showed that treatment with 150 mg dabigatran, twice a day, is not inferior to treatment with warfarin for prevention of recurrent VTE or VTE-related death. A total of 1,274 patients were randomized to receive dabigatran, 30 of whom developed recurrent VTE (2.4%), compared with 27 of 1,265 patients randomized to receive warfarin (2.1%). There was one VTE-related death in the group of patients treated with dabigatran (0.1%) and three deaths in the group of patients treated with warfarin (0.2%). Additionally, rates of major bleeding were similar in the two groups: 20 patients in the dabigatran group (1.6%) and 24 patients in the warfarin group (1.9%). In general, the frequency of bleeding was lower in the group given dabigatran than in the group treated with warfarin: 205 patients (16.1%) and 277 patients (21.7%) respectively. 28

### Rivaroxaban

 Rivaroxaban (Xarelto®) is an oral factor Xa inhibitor that binds reversibly to the site of activity of factor Xa. This medication has hepatic metabolization, an estimated half-life of 8 to 10 hours, and renal and fecal excretion. The EINSTEIN-DVT study compared standard treatment (enoxaparin followed by VKAs, warfarin, or acenocoumarol) of 1,718 patients with treatment of 1,731 patients with rivaroxaban (a total of 3,449 patients diagnosed with acute proximal DVT without symptomatic PE) over a 15-week period. The results demonstrated that administration of 15 mg of rivaroxaban, twice a day, for a period of 3 weeks, followed by administration of 20 mg for 12 weeks is not inferior to standard treatment for reduction of recurrent VTE. The incidence of recurrent VTE was 2.1% in the group treated with rivaroxaban compared with an incidence of 3.0% in the group given standard treatment. The principal outcome related to adverse effects — major and clinically relevant bleeding — was no more frequent, occurring in 139 patients (8.1%) in the group treated with rivaroxaban and 138 patients (8.1%) in the group treated with standard treatment. 29

### Apixaban

 Apixaban (Eliquis®) is a oral factor Xa inhibitor that impedes activity of factor Xa when free or bound to platelets, in a selective and reversible manner, and blocks activity of the prothrombinase complex. This medication has hepatic metabolization, plasma half-life from 8 to 15 hours, and renal and fecal excretion. The AMPLIFY study compared treatment of 2,704 patients with enoxaparin followed by warfarin with 2,691 patients treated with apixaban (a total of 5,395 patients) with symptomatic proximal DVT or PE (with or without DVT) for a 6-month period. The results demonstrate that treatment with 10 mg apixaban, twice a day, for 7 days, followed by 6 months of 5 mg apixaban, twice a day, is not inferior to treatment with enoxaparin followed by warfarin in terms of frequency of recurrent VTE and VTE-related mortality. However, the frequency of major bleeding was significantly lower in the group given apixaban (0.6%) than in the standard treatment group (1.8%). 30

### Edoxaban

 Edoxaban (Lixiana®) is an oral factor Xa inhibitor that bonds reversibly to the activity site of factor Xa. It has hepatic metabolization, plasma half-life of 9 to 11 hours, and 1/3 of excretion is renal and the remainder fecal. The phase III trial Hokusai-VTE compared treatment of 2,453 patients with heparin followed by VKAs (standard treatment) with treatment of 2,468 patients using edoxaban (a total of 4,921 patients) over a 12-month period. The patients eligible for this study had symptomatic acute DVT involving iliac, femoral, and popliteal veins or acute symptomatic PE (with or without DVT). The results showed that administration of 60 mg of edoxaban, once a day (or 30 mg of edoxaban for patients with creatinine clearance of 30-50 mL/min, body weight less than 60 kg, or receiving glycoprotein-P inhibitor) is not inferior to standard treatment in terms of recurrent VTE. The frequency of recurrent VTE was 3.2% (130 patients) in the group treated with edoxaban and 3.5% in the group treated with warfarin (146 patients). However, frequencies of major bleeding and clinically significant non-major bleeding were considerably lower in the group given edoxaban than in the group given warfarin: 8.5% (345 patients) and 10.3% (423 patients) respectively. 31 According to the laboratory that produces edoxaban, the medication is already being sold in South Korea, the United States, Japan, and some European countries. It was first approved in September 2014 in Japan, then in January 2015 in the United States, and in June 2015 in the European Union, and has also been approved in Hong Kong and Taiwan. In Brazil, the medication was approved in March 2018. 32

## CONCLUSIONS

 The available evidence suggests that treatment of DVT with DOACs, irrespective of the class, appears not to be inferior to standard treatment in terms of efficacy and safety. However, these medications are subject to certain restrictions, since the profile of adverse effects in children and patients with renal failure is unknown, and they are not yet indicated for treatment of cancer patients. 9 However, ongoing studies with 6-month follow-up, such as the Hokusai VTE Cancer, 33 Select-d, 34 and CARAVAGGIO studies 35 have demonstrated positive results for edoxaban, rivaroxaban, and apixaban, respectively, for treatment of patients with VTE and cancer. Furthermore, development of reversal agents has improved the safety profile of DOACs. Idarucizumab is available in Brazil under the commercial name Praxbind®, and andexanet alfa has also been approved for sale by the Food and Drug Administration (FDA), and it is believed that it will soon be approved by the National Agency for Sanitary Vigilance (Agência Nacional de Vigilância Sanitária - ANVISA). Ciraparantag is expected for the start of the next decade 36 and is intended to be a “universal” reversal agent for all anticoagulants other than warfarin. 

 It is undeniable that DOACs offer greater comfort for management of patients with VTE, both for physicians and their patients. This review reaffirms the robust evidence of the imminent capacity of DOACs to substitute medications used in conventional treatment. This enables us to reflect that we are close to a paradigm shift in anticoagulant treatment. Nevertheless, there is still a need for long-term studies that confirm that these new medications have safety profile that are not inferior to medications used in a standard treatment. There is still a long way to go before DOACs are consolidated as the definitive treatment for VTE. Currently, it is therefore necessary to take care when tailoring medical prescriptions to the peculiarities of the thromboembolic disease of each patient. 
