# Supplementary material for: Direct oral anticoagulants for treatment of deep vein thrombosis: overview of systematic reviews
Source: J Vasc Bras. 2018 Oct-Dec;17(4):310–7. doi: 10.1590/1677-5449.005518 (PMC6375259; doi:10.1590/1677-5449.005518)
Supplement: Translation

# Translation: Anticoagulantes orais diretos para o tratamento da trombose venosa profunda: revisão de revisões sistemáticas

**Authors:** Gustavo Muçouçah Sampaio Brandão, Raissa Carolina Fonseca Cândido, Hamilton de Almeida Rollo, Marcone Lima Sobreira, Daniela R. Junqueira

**Affiliations:** 1 Universidade Federal de São Carlos – UFSCar, Departamento de Medicina, Saúde do Adulto e Idoso - Cirurgia, São Carlos, SP, Brasil.; 2 Universidade Federal de Minas Gerais – UFMG, Centro de Estudos do Medicamento, Departamento de Farmácia Social, Faculdade de Farmácia, Belo Horizonte, MG, Brasil.; 3 Universidade Estadual Paulista – UNESP, Faculdade de Medicina de Botucatu, Departamento de Cirurgia e Ortopedia, Botucatu, SP, Brasil.; 4 University of Alberta, Edmonton, Canada.

**Keywords:** direct oral anticoagulants, deep venous thrombosis, systematic review

## Abstract

A number of limitations of standard therapy with warfarin for deep vein thrombosis (DVT) have been established. This overview of systematic reviews presents the baseline results for efficacy and safety of the new direct oral anticoagulants (DOACs) thrombin inhibitors, and activated factor X (Xa) inhibitors in patients with DVT. Searches were run on PubMed and the Cochrane Database of Systematic Reviews. Twenty-three studies were retrieved, and one systematic review was judged eligible. This review scored maximum according to AMSTAR criteria and included 7,596 patients for analysis of thrombin inhibitors and 16,356 patients for analysis of factor Xa inhibitors. The results of the meta-analysis indicate that DOACs are similar for DVT treatment when compared to standard treatment with warfarin. The incidence of major bleeding is somewhat lower in patients treated with factor Xa inhibitors and similar to standard therapy when treated with direct thrombin inhibitors.

## INTRODUÇÃO

 A trombose venosa profunda (TVP) dos membros inferiores é uma doença grave e potencialmente fatal. Sua incidência na população geral é de cinco casos por 10.000 habitantes por ano 1 . Aproximadamente, 46% dos casos de TVP proximal (TVP que atinge as regiões ílio-femoral, femoral e poplítea) podem evoluir para uma embolia pulmonar (EP), evento que, se não tratado, é fatal em 4% dos casos 2 . Além disso, complicações importantes, como a síndrome pós-trombótica, podem ocorrer em até 50% dos pacientes que sofrem uma TVP 3 . Assim, após a confirmação do diagnóstico de TVP, torna-se imperativo iniciar a terapia anticoagulante. O objetivo do tratamento é aliviar os sintomas, diminuir a extensão do trombo, a possibilidade de uma EP, impedir sua recorrência e atenuar a síndrome pós-trombótica. 

 O tratamento padrão se baseia, inicialmente, na administração parenteral de heparina não fracionada ou heparinas de baixo peso molecular durante 5 a 7 dias, seguida de terapia em longo prazo com antagonistas orais de vitamina K (AVKs) 4 ^,^ 5 . Os AVKs, como a varfarina, são tradicionalmente utilizados como anticoagulantes orais para tratamento e profilaxia do tromboembolismo venoso (TEV) desde a década de 1950 6 ^,^ 7 . Apesar de sua eficácia, a varfarina é limitada por fatores como interações medicamentosas, interações alimentares, início de ação lento, risco de hemorragia, alopecia, necrose de pele e necessidade de monitoramento rigoroso para manter a relação normalizada internacional (RNI) dentro do índice terapêutico. Essas limitações impulsionaram o desenvolvimento de pesquisas com novos anticoagulantes que, idealmente, deveriam apresentar risco de sangramento reduzido, ocorrência reduzida de efeitos colaterais, ausência de interação com outros medicamentos e alimentos, ser de fácil administração, permitir o tratamento domiciliar, não necessitar de controle laboratorial, possuir custo acessível e apresentar um antídoto para reverter a anticoagulação nos casos de sangramentos extensos e clinicamente relevantes 8 . 

### Anticoagulantes orais diretos

 De acordo com as diretrizes do *American College of Chest Physician* 9 (ACCP), duas formas de anticoagulantes orais são indicadas para o tratamento da TVP: os inibidores diretos da trombina e os inibidores do fator Xa. 

 Os inibidores diretos da trombina, como a dabigatrana, ligam-se diretamente à trombina sem a necessidade de um cofator, como a antitrombina. Diferentemente dos AVKs e das heparinas, os inibidores diretos da trombina podem inibir tanto a trombina solúvel quanto a trombina ligada à fibrina 10 . Por não se ligarem a outras proteínas, os inibidores diretos da trombina apresentam poucas limitações farmacocinéticas e farmacodinâmicas, o que torna a resposta anticoagulante mais previsível. Além disso, os inibidores diretos da trombina não apresentam efeito antiplaquetário e não induzem trombocitopenia induzida pela heparina 11 . 

 Os inibidores do fator Xa se ligam diretamente ao sítio ativo do fator Xa, bloqueando a atividade desse fator de coagulação. Diferentemente dos pentassacarídeos (inibidores indiretos do fator Xa), esses medicamentos inativam o fator Xa livre e o fator Xa incorporado ao complexo protrombinase, além de não interagirem com o inibidor antitrombínico 12 . Os inibidores do fator Xa indicados para o tratamento da TVP são: rivaroxabana, apixabana e edoxabana. 

 A utilização clínica efetiva e segura dos anticoagulantes orais diretos (DOACs) requer o acompanhamento das evidências sobre a eficácia clínica desses medicamentos. O estudo dos efeitos adversos, principalmente sangramento, também é essencial para compreender o balanço de benefícios e efeitos nocivos em comparação aos anticoagulantes atualmente utilizados na prática clínica. Portanto, nosso objetivo foi revisar as evidências científicas sobre os benefícios e efeitos adversos dos novos anticoagulantes no tratamento da TVP em comparação com a terapia padrão (heparina de baixo peso molecular ou heparina não fracionada seguida por AVKs). 

## MÉTODOS

 Revisão de revisões sistemáticas (*overview of systematic reviews*) sobre a eficácia e os efeitos adversos do tratamento de pacientes com TVP com os DOACs, de modo que foram incluídos estudos de revisão sistemática de ensaios clínicos controlados e randomizados comparando a terapia padrão com DOACs em pacientes diagnosticados com TVP. Não foram aplicados limites de ano nem de idioma da publicação. Revisões narrativas, *guidelines* e opiniões de especialistas não foram consideradas. 

 A busca foi realizada nas bases de dados PubMed e *Cochrane Database of Systematic Reviews* . Na base de dados PubMed, foi utilizado um filtro de busca altamente sensível para a recuperação de revisões sistemáticas 13 . A estratégia de busca nas duas bases de dados utilizou uma combinação dos termos com o respectivo símbolo de truncagem: novel oral anticoagulant* and deep vein thrombosis. 

 A triagem dos estudos recuperados foi conduzida de forma independente por dois autores. A triagem inicial consistiu da leitura de todos os títulos e resumos dos estudos recuperados, e os artigos completo dos estudos considerados potencialmente elegíveis nessa etapa foram analisados posteriormente. Os conflitos do processo de seleção foram resolvidos com a participação de um terceiro autor. 

 Dois desfechos foram considerados essenciais para avaliar a eficácia e os efeitos adversos dos DOACs em relação aos AVKs: (i) recorrência de TVP ou TEV; e (ii) sangramento. Dados sobre esses desfechos e de descrição geral dos estudos foram extraídos por um autor e revisados por um segundo autor. Os dados foram extraídos de acordo com um formulário de extração de dados, descrevendo os estudos de acordo com a data da atualização da busca sistemática, população de pacientes estudada, o tipo de anticoagulante oral estudado e a quantidade de ensaios clínicos incluídos. 

 A validade interna (qualidade metodológica) das revisões sistemáticas foi avaliada com o uso da ferramenta AMSTAR 14 ^,^ 15 . AMSTAR é uma ferramenta validada composta por 11 itens de respostas diretas (sim; não; não posso responder; não aplicável) que avalia, entre outras coisas, a existência de planejamento a priori da revisão sistemática, se a seleção e extração de dados foram realizadas em duplicata e se o status da publicação foi utilizado como critério de inclusão. A qualidade da revisão pode ser analisada de acordo com a pontuação final atingida (máximo 11). 

 As revisões sistemáticas incluídas foram descritas em termos de suas características gerais (por exemplo, população de pacientes, medicamentos estudados), resultados sobre os desfechos de interesse e sua qualidade metodológica. Uma análise estatística (metanálise) não pôde ser realizada devido ao limitado número de estudos incluídos. 

## RESULTADOS

 No total, 23 artigos foram recuperados. Após a exclusão de duplicados, 21 artigos foram avaliados de acordo com os critérios de inclusão. Desses, 19 artigos foram excluídos com base na leitura do título e resumo, e dois artigos que foram considerados potencialmente elegíveis tiveram o artigo completamente analisados. Ao final do processo de seleção, uma revisão sistemática foi considerada elegível e incluída em nossa revisão ( Figura 1 ). 

**Figura 1:** Diagrama de identificação e seleção dos artigos para revisão sistemática comparando terapia padrão da trombose venosa profunda com novos anticoagulantes orais.

 A revisão sistemática conduzida por Robertson e colaboradores 16 comparou o tratamento com inibidores diretos de trombina e do fator Xa com terapia padrão. Todos os inibidores de trombina e do fator Xa foram comparados com um grupo, e não foi realizada comparação individualizada para cada medicamento. A comparação relativa aos inibidores de trombina incluiu um estudo do medicamento ximelagran, retirado do mercado em 2006 devido ao relato de dano hepático grave durante tratamento continuado (mais de 11 dias) 17 . 

 A revisão atingiu escore máximo no AMSTAR ( Tabela 1 ), e incluiu 7.596 pacientes no grupo de pacientes para análise dos inibidores diretos de trombina e 16.356 pacientes para a análise dos inibidores diretos do fator Xa ( Tabela 2 ). Os resultados da metanálise realizada na revisão sistemática indicaram similaridade de eficácia na prevenção do tromboembolismo venoso e de incidência de sangramento maior (hemorragia grave, conforme definição da Sociedade Internacional de Trombose e Hemostasia – ISTH 18 ) entre a terapia padrão e os anticoagulantes orais da classe de inibidores diretos da trombina. Os DOACs inibidores do fator Xa também apresentaram eficácia semelhante à terapia padrão. A incidência de sangramento maior foi um pouco menor nos pacientes que estavam recebendo os novos anticoagulantes orais inibidores do fator Xa. 

**Tabela 1:** Descrição da revisão sistemática comparando terapia padrão da trombose venosa profunda com novos anticoagulantes orais.

**Autor, Ano**	**Atualização da busca**	**População**	**Anticoagulante oral**	**Ensaios clínicos incluídos (n)**	**AMSTAR**
Robertson, 2015	Janeiro 2017	Pacientes com diagnóstico de trombose venosa profunda confirmado por técnica padrão de imagem (venografia, impedância pletismográfica, ultrassonografia de compressão distal, ultrassonografia de compressão proximal)	Inibidores diretos da trombina e Inibidores diretos do fator Xa	3 ensaios clínicos comparando inibidores diretos da trombina com terapia padrão; 8 ensaios clínicos comparando inibidores diretos do fator Xa com terapia padrão	11/11

**Tabela 2:** Recorrência de tromboembolismo venoso e incidência de sangramento maior em pacientes com trombose venosa profunda recebendo tratamento com novos anticoagulantes orais e comparação com terapia padrão.

**Comparação**	**Pacientes**	**Recorrência de trombose venosa profunda ou embolismo venoso**	**Sangramento maior**	**Qualidade dos estudos contribuindo para a metanálise**
		**(OR, IC95%)**		
Inibidores diretos da trombina (ximelagran, dabigatran)	7.596 pacientes, idade média (min 54,7, máx. 57,1)	≤ 3 meses: OR 1,09 (IC95% 0,62-1,91);	≤ 3 meses: (OR 0,54; IC95% 0,28-1,03);	Julgamento dos autores: “*We deemed all included studies to be of high methodological quality and generally low risk of bias*.”
		> 3 meses: OR 1,09 (IC95% 0,76-1,58);	> 3 meses: (OR 0,76; IC95% 0,49-1,18);	
		6 meses: 1,09 (IC95% 0,80-1,49)	6 meses: OR 0,68 (IC95% 0,47-0,98)	
Inibidores diretos do fator Xa (apixaban, rivaroxaban, edoxaban)	16.356 pacientes idade média (mín. 53,1, máx. 60)	≤ 3 meses: OR 0,69 (IC95% 0,48-0.99);	≤ 3 meses: OR 0,83 (IC95% 0,47-1.45);	Julgamento dos autores: “*We deemed all included studies to be of high methodological quality and generally low risk of bias*.”
		> 3 meses: OR 0,97 (IC95% 0,78-1,22);	> 3 meses: OR 0,50 (IC95% 0,36-0,71);	
		6 meses: 0,89 (IC95% 0,73-1,07)	6 meses: OR 0,57 (IC95% 0,43-0,76)	

OR: odds ratio; IC: intervalo de confiança.

## DISCUSSÃO

 As evidências acumuladas indicam que os DOACs, tanto inibidores diretos de trombina quanto do fator Xa, apresentam um balanço de eficácia e efeitos adversos semelhantes ao da terapia padrão. Isso significa que esses medicamentos não apresentam balanço entre risco e benefício diferente do apresentado pela combinação de heparinas com AVKs no tratamento da TVP. 

 As evidências encontradas são limitadas a uma revisão sistemática. A revisão sistemática disponível para essa avaliação foi considerada de alta qualidade metodológica. No entanto, a probabilidade de seus resultados serem alterados por novas pesquisas depende da qualidade metodológica dos ensaios clínicos incluídos e analisados na revisão sistemática. Infelizmente, a revisão sistemática em questão não realizou uma avaliação da qualidade das evidências segundo a metodologia GRADE (*Grading of Recommendations, Assessment, Development and Evaluations* ) 19 ^-^ 21 , o que permitiria inferir com mais segurança o grau de qualidade dessas evidências com as recomendações clínicas praticadas. O número de pacientes incluídos na análise quantitativa dos desfechos recorrência de TVP ou EP e sangramento maior incluíram um número relevante de pacientes. No entanto, os ensaios clínicos randomizados incluídos nessa revisão sistemática parecem ter sua qualidade reduzida devido a risco de viés, principalmente em relação à geração da sequência de alocação randômica do tratamento. Assim, apesar das conclusões parecerem consistentes, evidências adicionais aumentarão a confiabilidade dos dados para informar decisões clínicas definitivas. 

 Uma limitação desses resultados é o fato de a análise de eficácia e de efeitos adversos ter sido realizada somente para o grupo de medicamentos das duas categorias de DOACs, inibidores diretos da trombina e do fator Xa. Apesar de ser provável que medicamentos individuais que compartilham do mesmo mecanismo de ação apresentem efeitos semelhantes, é útil a comprovação empírica do efeito clínico de cada medicamento. 

 Os DOACs apresentam maior facilidade no gerenciamento da terapia anticoagulante, são administrados em doses fixas e sem necessidade de ajuste pelo peso corporal. Esses medicamentos apresentam um início de ação rápido, não necessitam de controle laboratorial e apresentam meia-vida curta (o que facilita o manejo dos pacientes quando há necessidade de suspensão dos medicamentos para realização de um procedimento diagnóstico ou cirúrgico). As interações fármaco-fármaco, fármaco-álcool e fármaco-alimentos descritas até o momento são poucas e permitem o tratamento ambulatorial. 

 Um problema prático da utilização dos DOACs, no entanto, está relacionado às diferentes terapias, doses e, principalmente, posologias desses medicamentos. 

 Isso porque, enquanto alguns são utilizados como monoterapia (por exemplo, rivaroxabana, apixabana), outros (por exemplo, dabigatrana, edoxabana) são utilizados como terapia adjuvante a heparina de baixo peso molecular. Além disso, mesmo os medicamentos utilizados como monoterapia apresentam diferentes doses e posologias, as quais podem variar durante a fase inicial e a fase de manutenção da anticoagulação (por exemplo, rivaroxabana). A terapia padrão, estabelecida como prática clínica há mais de 50 anos, apresenta um esquema terapêutico mais eficaz, seguro e compatível com o atendimento em nível ambulatorial, apesar dos ajustes de doses necessários. Outro problema prático relevante é o acesso a esses novos medicamentos, especialmente em países com baixo nível de desenvolvimento socioeconômico, uma vez que apresentam custo elevado. 

 Um fator determinante para a utilização clínica rotineira dos DOACs é a disponibilidade de um agente reversor disponível para toda essa nova classe de medicamentos. O efeito anticoagulante dos AVKs pode ser revertido pela administração de vitamina k, plasma fresco congelado ou complexo protrombínico 22 . Nos Estados Unidos, o idarucizumab, agente reversor específico para a dabigatrana, já foi aprovado para uso clínico. Trata-se de um fragmento de anticorpo monoclonal que possui maior afinidade à dabigatrana quando comparado à trombina e, portanto, reverte seu efeito coagulante 23 . E, recentemente, também foi aprovado o andexanet alfa, uma molécula de fator Xa recombinante que pode se ligar tanto aos inibidores diretos do fator Xa (como rivaroxabana, apixabana e edoxabana), quanto aos inibidores do fator Xa que necessitam da ação da antitrombina (como às heparinas de baixo peso molecular e fondaparinux) 24 ^,^ 25 . No Brasil, apenas o idarucizumab já possui autorização para uso e comercialização 26 . 

 A seguir, apresentamos uma revisão sumarizada do *status* dos estudos de fase III disponíveis sobre os diferentes DOACs. 

### Dabigatrana

 O etexilato de dabigatrana (Pradaxa®) é um pró-fármaco rapidamente metabolizado pelo fígado, transformando-se em um composto ativo que se liga de forma competitiva e reversível ao sítio de atividade da trombina, bloqueando sua atividade pró-coagulante. A dabigatrana é absorvida pelo trato gastrointestinal, apresenta uma meia-vida de 12 a 17 horas, e excreção renal e fecal. O estudo RE-COVER comparou o tratamento da varfarina com a dabigratrana, após o tratamento inicial com um anticoagulante parenteral, em 2.539 pacientes diagnosticados com TEV agudo pelo período de 6 meses 27 . Os resultados mostraram que o tratamento com dabigatrana 150 mg, duas vezes ao dia, não é inferior ao tratamento com varfarina na prevenção do TEV recorrente ou morte relacionada ao TEV. Entre os 1.274 pacientes randomizados para receber dabigatrana, um total de 30 desenvolveram TEV recorrente (2,4%), comparados com 27 de 1.265 pacientes randomizados para receber varfarina (2,1%). Houve uma morte relacionada a TEV no grupo de pacientes tratados com a dabigatrana (0,1%) e três mortes no grupo de pacientes tratados com a varfarina (0,2%). Além disso, as taxas de sangramento maior foram semelhantes nos dois grupos: 20 pacientes no grupo da dabigatrana (1,6%) e 24 pacientes no grupo da varfarina (1,9%). No geral, a frequência de sangramento foi menor no grupo que recebeu dabigatrana em relação ao grupo tratado com varfarina: 205 pacientes (16,1%) e 277 pacientes (21,7%) respectivamente 28 . 

### Rivaroxabana

 A rivaroxabana (Xarelto®) é um inibidor oral do fator Xa que se liga de forma reversível ao sítio de atividade do fator Xa. O medicamento apresenta metabolização hepática, meia-vida estimada entre 8 e 10 horas e excreção renal e fecal. O estudo EINSTEIN-DVT comparou o tratamento padrão (enoxaparina seguida por AVKs, varfarina ou acenocoumarol) realizado em 1.718 pacientes com o tratamento realizado em 1.731 pacientes com rivaroxabana (total de 3.449 pacientes diagnosticados com TVP aguda proximal sem EP sintomática) pelo período de 15 semanas. Os resultados demostraram que a administração de 15 mg de rivaroxabana, duas vezes ao dia, pelo período de 3 semanas, seguida da administração de 20 mg por 12 semanas não é inferior à terapia padrão para a redução de TEV recorrente. A incidência de TEV recorrente foi de 2,1% no grupo tratado com rivaroxabana em comparação a uma incidência de 3,0% do grupo que recebeu a terapia padrão. O principal desfecho relacionado aos efeitos adversos — sangramento maior e clinicamente relevante não maior — ocorreu em 139 pacientes (8,1%) do grupo tratado com rivaroxabana e 138 pacientes (8,1%) do grupo tratado com terapia padrão 29 . 

### Apixabana

 A apixabana (Eliquis®) é um inibidor oral do fator Xa que impede a atividade do fator Xa livre ou ligado a plaqueta, de forma seletiva e reversível, e bloqueia a atividade do complexo protrombinase. O medicamento apresenta metabolização hepática, meia-vida plasmática de 8 a 15 horas e excreção renal e fecal. O estudo AMPLIFY comparou o tratamento de 2.704 pacientes usando enoxaparina seguida por varfarina com 2.691 pacientes usando apixabana (total de 5.395 pacientes) com TVP sintomática proximal ou EP (com ou sem TVP) pelo período de 6 meses. Os resultados demostraram que o tratamento com apixabana 10 mg, duas vezes ao dia, por 7 dias, seguido de 6 meses de apixabana 5 mg, duas vezes ao dia, não é inferior ao tratamento com enoxaparina seguido pela varfarina em relação à frequência de TEV recorrente e de mortalidade relacionada ao TEV. No entanto, a frequência de sangramento maior foi significativamente menor no grupo que recebeu apixabana (0,6%) em comparação ao grupo tratado com a terapia padrão (1,8%) 30 . 

### Edoxabana

 A edoxabana (Lixiana®) é um inibidor oral do fator Xa que se liga de forma reversível ao sítio de atividade do fator Xa. Apresenta metabolização hepática, meia-vida plasmática de 9 a 11 horas, 1/3 de excreção renal e o restante fecal. O estudo de fase III Hokusai-VTE comparou o tratamento de 2.453 pacientes usando heparina seguida por AVKs (tratamento padrão) com o tratamento de 2.468 pacientes usando edoxabana (total de 4.921 pacientes) pelo período de 12 meses. Os pacientes elegíveis para esse estudo apresentavam TVP aguda sintomática envolvendo as veias ilíaca, femoral e poplítea ou EP aguda sintomática (com ou sem TVP). Os resultados mostraram que a administração de 60 mg de edoxabana, uma vez ao dia (ou 30 mg de edoxabana em pacientes com *clearence* de creatinina entre 30-50 mL/min, peso corporal menor que 60 kg ou recebendo inibidor da glicoproteína-P) não é inferior ao tratamento padrão em relação ao TEV recorrente. A frequência de TEV recorrente foi igual a 3,2% (130 pacientes) no grupo tratado com edoxabana e igual a 3,5% no grupo tratado com varfarina (146 pacientes). No entanto, as frequências de sangramento maior e sangramento clinicamente significante não maior foram expressivamente menores no grupo que recebeu edoxabana em relação ao grupo que recebeu varfarina: 8,5% (345 pacientes) e 10,3% (423 pacientes) respectivamente 31 . Segundo o laboratório que produz a edoxabana, o medicamento já está sendo comercializado na Coreia do Sul, Estados Unidos, Japão e em alguns países da Europa. Sua primeira aprovação ocorreu em setembro de 2014 no Japão, seguida pela aprovação em janeiro de 2015 nos Estados Unidos e em junho de 2015 na Comissão Europeia, tendo sido aprovado também em Hong Kong e Taiwan. No Brasil, o medicamento foi aprovado em março de 2018 32 . 

## CONCLUSÃO

 As evidências disponíveis sugerem que o tratamento da TVP com os DOACs, independentemente da classe, parece ser não inferior à terapia padrão em termos de eficácia e segurança. No entanto, esses medicamentos apresentam algumas importantes restrições, pois não se conhece o perfil de efeitos adversos em crianças e em pacientes com insuficiência renal, além de ainda não serem indicados para o tratamento de pacientes oncológicos 9 . Porém, estudos em andamento com seguimento de 6 meses, como os estudos Hokusai VTE Cancer 33 , Select-d 34 e CARAVAGGIO 35 , têm demonstrado resultados positivos para edoxabana, rivaroxabana e apixabana, respectivamente, quanto ao tratamento de pacientes com TEV associado ao câncer. Além disso, o desenvolvimento dos agentes reversores tem aumentado o perfil de segurança dos DOACs. O idarucizumab se encontra disponível no Brasil com o nome comercial Praxbind ^®^, e o andexanet alfa também foi aprovado para comercialização pela Food and Drug Administration (FDA), acreditando-se que em breve possa estar liberado pela Agência Nacional de Vigilância Sanitária (ANVISA). Espera-se, para o começo da próxima década, o ciraparantag 36 , com a proposta de ser um reversor “universal” dos anticoagulantes, exceto varfarina. 

 É inegável que os DOACs trouxeram conforto no manejo dos pacientes com TEV, tanto para os médicos quanto para os pacientes. Esta revisão reforça as robustas evidências da iminente capacidade dos DOACs em substituir os medicamentos usados no tratamento convencional. Isso permite refletir que estamos próximos a uma mudança de paradigma na terapia anticoagulante. Contudo, ainda são necessários estudos de longo prazo que confirmem a não inferioridade do perfil de segurança desses novos medicamentos em relação aos medicamentos usados na terapia padrão. Há, portanto, um longo caminho a ser percorrido antes dos DOACs se firmarem como terapia definitiva do TEV. Assim, nesse momento, é necessário haver cautela e individualizar a prescrição médica conforme as peculiaridades da doença tromboembólica de cada paciente.
